# The impact of the motherhood role on recovery motivation in women with severe mental illness

**DOI:** 10.1080/07853890.2026.2714593

**Published:** 2026-08-18

**Authors:** Menekşe Sıla Yazar, Eren Yıldızhan, Kumru Şenyaşar Meterelliyoz, Selim Arpacıoğlu, Erkan Aydın

**Affiliations:** ^a^ School of Medicine, Psychiatry Department, Altınbaş University, Istanbul, Turkey; ^b^ Bakırköy Prof Dr. Mazhar Osman Mental Health and Neurological, Diseases Education and Research Hospital, Turkey; ^c^ Bağcılar Research and Training Hospital, Psychiatry Clinic, Istanbul, Turkey

**Keywords:** Bipolar disorder, schizophrenia, motherhood, recovery motivation, parental role, ınsight

## Abstract

**Objective:**

This study aimed to examine the association between maternal role perceptions and subjective recovery motivation among women with severe mental illness and to explore whether these relationships differ between schizophrenia and bipolar disorder (BD).

**Methods:**

In this cross-sectional study, 115 mothers with a schizophrenia diagnosis (*n* = 40) or bipolar disorder (*n* = 75) who were receiving services from community mental health centres were included. Maternal role perceptions and subjective recovery motivation were evaluated using the Self-Perception of Parental Role Scale (SPPR) and the Subjective Recovery Assessment Scale (SubRAS), respectively. The evaluation of psychiatric symptom severity, functional status, and cognitive insight was performed by using standardized clinical instruments, including the Brief Psychiatric Rating Scale (BPRS) and Personal and Social Performance Scale (PSP).

**Results:**

Women with BD demonstrated a higher median SubRAS score compared with those with schizophrenia (62 vs. 54). A significant positive relationship was observed between subjective recovery motivation and SPPR role satisfaction (*r* = 0.296), parental efficacy (*r* = 0.276), parental investment (*r* = 0.331), role balance (*r* = 0.240), and total SPPR score (*r* = 0.413) (all FDR-adjusted *p* < 0.05). Recovery scores were also positively associated with functioning (PSP: *r* = 0.463) and cognitive insight (*r* = 0.320), while negatively correlated with psychiatric symptom severity (BPRS: r=–0.394). Multivariable regression analyses revealed that parental investment was an independent predictor of recovery motivation (*p* = 0.049), with lower BPRS scores showing an association with higher recovery scores (*p* = 0.028).

**Conclusion:**

Positive perceptions of the maternal role are associated with greater subjective recovery motivation among women with severe mental illness. These findings highlight the potential importance of incorporating parenting roles and maternal identity into recovery-oriented mental health services.

## Introduction

Community-based services have increasingly shifted from institutional treatment for those with severe mental illness over the past several decades. Beyond influencing treatment modalities, this transformation has also reshaped the social and familial context for individuals with psychiatric disorders. Despite these changes, relatively limited attention has been given to the parental experiences of individuals with severe mental illness, particularly mothers who must balance psychiatric challenges with caregiving responsibilities [1]. Most previous studies have focused on the outcomes and vulnerabilities of children of parents with mental illness. In contrast, fewer studies have examined the influence of psychiatric disorders on parental responsibilities and the formation of parental identity [1,2].

Motherhood plays an important social and psychological role for many women and frequently forms a central component of personal identity. Nevertheless, women diagnosed with severe mental health conditions face significant social stigma related to parenting, including concerns regarding caregiving capacity, perceived risk of harm to children and potential child custody issues [3]. The stigma associated with these issues may contribute to increased scrutiny from social service organizations and may affect treatment engagement and interactions with mental health systems [1]. Recent studies increasingly describe motherhood as a meaningful and motivating life role [4].

Within recovery-oriented mental health care, meaningful life roles are considered central to personal recovery. The concept of recovery has expanded beyond symptom reduction to include the development of identity, purpose, hope, and empowerment despite ongoing mental health challenges [5]. The CHIME framework conceptualizes recovery as a multidimensional process encompassing connectedness, hope, identity, meaning, and empowerment, suggesting that socially valued roles such as parenting may support several of these domains [5,6]. In this situation, motherhood might serve as a crucial way for identity reconstruction and personal meaning for women with severe mental illness.

The relationship between motherhood and mental health illness is multifaceted, presenting a spectrum of both supportive and challenging experiences. The responsibilities of parenthood could provide emotional fulfillment and motivation for recovery, though they might concurrently engender additional stressors related to caregiving demands, financial strain, and social stigma [2,7]. Previous studies indicated that parental responsibilities could both facilitate and challenge recovery, underscoring the importance of a more thorough examination of how maternal roles affect recovery outcomes for women with severe mental illness [2,7].

Even with increasing attention to recovery-oriented care, relatively few studies have investigated how certain aspects of a maternal role perception relate to recovery motivation especially in women with severe mental illness [2,8]. A comprehensive understanding of these relationships could provide important insights into the impact of meaningful social roles on recovery and may guide the development of psychosocial interventions for supporting parental identities in mental health services.

The present study aimed to investigate the association between perceived motherhood role and subjective recovery motivation among women with severe mental illness. Specifically, we explored whether different dimensions of maternal role perception—including parental investment, competence, satisfaction, and role balance—were associated with recovery motivation and whether these relationships differed between women with schizophrenia (SCH) and those with BD. In accordance with recovery-oriented frameworks and prior research on parenting and mental illness, we hypothesized that more positive perceptions of the parental role would be associated with higher levels of subjective recovery motivation. Specifically, we expected that maternal role investment and competence would show stronger associations with recovery motivation among women with SCH, whereas parental role satisfaction might be more strongly related to recovery motivation among women with BD.

## Methods

### Participants and procedure

This cross-sectional study included mothers aged between 18 and 65 years who had been diagnosed with SCH or BD according to the Diagnostic and Statistical Manual of Mental Disorders, Fifth Edition (DSM-5) [9].

Participants were recruited from community mental health centres (CMHCs) in Güngören and Bağcılar, Istanbul, Türkiye, which are affiliated with the Ministry of Health and operate in connection with the Istanbul Bakırköy Prof. Dr. Mazhar Osman Mental Health and Neurological Diseases Training and Research Hospital. In Türkiye, CMHCs provide multidisciplinary community-based mental health services including psychiatric follow-up, psychosocial rehabilitation, and family support for individuals with severe mental illness.

The study included individuals who had a confirmed diagnosis of SCH or BD, were in clinical remission for at least six months, and had received psychiatric treatment for at least one year.

Participants with active psychotic, manic, or depressive episodes, a history of alcohol or substance use disorders, or intellectual disabilities that could interfere with study participation were excluded from the study.

Eligible participants attending routine outpatient visits at the CMHCs were informed about the study and invited to participate. All participants provided written informed consent prior to inclusion in the study. This study adhered to the Declaration of Helsinki, with approval from the Ethics Committee of Bakırköy Prof. Dr. Mazhar Osman Mental Health and Neurological Diseases Training and Research Hospital (Decision No: 329, 23 July 2019).

### Data collection procedures

Data were collected through face-to-face clinical interviews conducted by experienced clinicians. Sociodemographic information, clinical characteristics, and motherhood-related experiences were recorded using a structured data collection form developed by the researchers.

The form collected standard demographic information, including age, marital status, education level, and economic status, and included three additional questions designed to explore motherhood-related experiences in the context of mental illness. Participants were asked whether they had ever been involved in a child custody case, whether motherhood influenced their adherence to treatment, and whether motherhood had a positive, neutral, or negative effect on coping with mental illness.

The evaluation of clinical symptom severity, functioning, cognitive insight, parental role perceptions, and subjective recovery was performed *via* validated psychiatric and psychometric instruments. Detailed descriptions of the scales used in the study are provided in Supplementary Table S1.

## Measures

### Self-perception of parental role scale (SPPR)

Maternal role perceptions were evaluated utilizing the SPPR [10]. The SPPR, a 22-item self-report instrument, evaluates parents’ perceptions of their role through four dimensions: parental competence (6 items), parental satisfaction (6 items), parental investment (5 items), and role balance (5 items). A Likert-type scale was employed for item evaluation; higher numerical values correlate with more positive impressions of the parenting role. This scale has been a common tool in studies investigating parental identity and role perceptions.

### Subjective recovery assessment scale (SubRAS)

Subjective recovery motivation was evaluated using the SubRAS, developed and validated in a Turkish population of individuals with SCH [11]. The SubRAS is a 17-item self-report scale designed to evaluate subjective aspects of personal recovery. The assessment uses a 5-point Likert scale for each item, resulting in a total score ranging from 17 to 85; with higher scores reflecting greater levels of perceived recovery.

### Brief psychiatric rating scale (BPRS)

Assessment of general psychiatric symptom severity was conducted with the BPR, a clinician-rated instrument developed by Overall and Gorham [12]. The BPRS includes 18 items designed to evaluate symptoms like depression, hallucinations, anxiety, and emotional withdrawal, each rated on a 7-point scale. Higher scores indicate greater psychiatric symptom severity.

### Positive and negative syndrome scale (PANSS)

PANSS was used to assess symptom severity in participants diagnosed with SCH or other psychotic disorders [13]. The PANSS, a 30-item assessment, evaluates positive symptoms, negative symptoms, and general psychopathology. Each item is scored from 1 to 7, with higher scores representing more severe symptomatology.

### Personal and social performance scale (PSP)

Social and occupational functioning was assessed using the PSP [14]. The PSP provides an evaluation of functionality within four distinct domains: socially useful activities, personal and social relationships, self-care, and aggressive behaviour. Scores range from 1 to 100, with higher scores indicating better functioning.

### Beck cognitive insight scale (BCIS)

BCIS, a 15-item self-report tool, was utilized to evaluate cognitive insight by assessing an individual’s capacity for self-reflection and questioning their own beliefs [15]. The scale includes two subdimensions: self-reflectiveness and self-certainty. Higher total scores indicate greater cognitive insight.

### Hamilton depression rating scale (HDRS)

The HDRS, a clinician-rated scale developed by Hamilton [16], was employed to evaluate depressive symptom severity in individuals diagnosed with BD. The HDRS, a 17-item questionnaire, evaluates the intensity of depressive symptoms severity, with higher scores indicating more severe depression.

### Young mania rating scale (YMRS)

The severity of manic symptoms in BD was assessed using the YMRS, an 11-item clinician-rated scale [17]. Total scores range from 0 to 44, with higher scores indicating greater mania severity.

## Statistical analysis

Statistical analyses were performed using the Statistical Package for the Social Sciences (SPSS), version 23.0 (IBM Corp., Armonk, NY, USA). The distribution of continuous variables was evaluated using the Kolmogorov–Smirnov test. Continuous variables were expressed as median (minimum–maximum), while categorical variables were summarized as frequencies and percentages. Comparisons between two independent groups were conducted using the Mann–Whitney U-test for continuous variables. Categorical variables were compared using Pearson’s chi-square test or Fisher’s exact test, as appropriate.

All statistical tests were two-sided, and a *p*-value <0.05 was considered statistically significant. Given the relatively large number of comparisons performed in correlation analyses and group comparisons, the false discovery rate (FDR) was controlled using the Benjamini–Hochberg procedure. This approach adjusts *p*-values while maintaining an acceptable proportion of expected false positive findings among statistically significant results. Accordingly, results from correlation analyses and group comparisons are presented as FDR-adjusted p-values. In contrast, no multiplicity correction was applied to regression models, as these analyses were designed to identify independent predictors within a multivariable framework rather than to test multiple independent hypotheses.

Associations between continuous variables were evaluated using Spearman’s rank correlation analysis.

To identify factors independently associated with subjective recovery (SubRAS scores), multivariable linear regression analyses were performed. Sociodemographic characteristics, functioning scores, clinical symptom severity measures, and parental role dimensions were considered as candidate predictors. Separate regression models were constructed for the overall sample and for diagnostic subgroups.

Model selection was performed using a backward elimination procedure guided by the Akaike Information Criterion (AIC). In this approach, predictors were sequentially removed from the full model, and models were compared based on their AIC values. The AIC evaluates the trade-off between model goodness-of-fit and model complexity, penalizing models with excessive numbers of parameters to reduce the risk of overfitting. The model with the lowest AIC value was selected as the most parsimonious model providing the optimal balance between explanatory power and model simplicity.

Regression results were reported as unstandardized coefficients (B), standardized beta coefficients, 95% confidence intervals, and adjusted R^2^ values indicating the proportion of explained variance. Multicollinearity among predictors was assessed using variance inflation factors (VIF). In addition, sensitivity analyses using alternative model specifications with different combinations of predictors were conducted to examine the robustness of the regression findings.

## Results

The study encompassed 115 women with severe mental illness; 40 (34.8%) were identified as having SCH, and 75 (65.2%) BD. The median age of the participants in the study was 44.0 years (23.0–66.0). The demographic, clinical, and motherhood-related characteristics at baseline, stratified by diagnosis, are detailed in Table 1.

**Table 1.** Demographic, clinical, and motherhood-related characteristics by diagnosis (schizophrenia vs bipolar disorder).

Variable	*n*	Total	Schizophrenia	Bipolar disorder	Raw *p* [2]	FDR-adjusted *p* [3]	Effect size [4]
		*N* = 115 [1]	*N* = 40 [1]	*N* = 75 [1]			
**Age (years)**	115	44.0 (23.0–66.0)	44.0 (25.0–66.0)	45.0 (23.0–64.0)	0.386	0.695	0.448
**Duration of illness (years)**	115	17.0 (3.0–38.0)	18.0 (4.0–35.0)	15.0 (3.0–38.0)	**0.019**	0.102	0.280
**Number of children**	115	2.0 (1.0–10.0)	2.0 (1.0–4.0)	2.0 (1.0–10.0)	0.649	0.835	0.497
**Age of children (years)**	115	18.0 (0.0–42.0)	19.0 (2.0–42.0)	18.0 (0.0–40.0)	0.495	0.799	0.469
**SPPR**							
*Role Satisfaction*	115	21.0 (7.0–25.0)	21.0 (7.0–25.0)	20.0 (9.0–25.0)	0.510	0.799	0.472
*Parental Efficacy*	115	21.0 (7.0–30.0)	19.0 (8.0–29.0)	21.0 (7.0–30.0)	0.253	0.526	0.676
*Parental Investment*	115	15.0 (5.0–26.0)	15.5 (5.0–25.0)	14.0 (10.0–26.0)	0.770	0.945	0.513
*Role Balance*	115	21.0 (8.0–30.0)	22.0 (10.0–30.0)	21.0 (8.0–30.0)	0.939	>0.999	0.538
*SPPR Total Score*	115	76.0 (41.0–106.0)	76.0 (41.0–95.0)	76.0 (47.0–106.0)	0.977	>0.999	0.550
**SubRAS Total**	115	59.0 (20.0–87.0)	54.0 (23.0–84.0)	62.0 (20.0–87.0)	**0.013**	0.089	0.828
**Clinical Global Impression**	39	3.0 (2.0–4.0)	3.0 (2.0–4.0)	3.0 (3.0–3.0)	0.848	0.994	0.132
**Brief Psychiatric Rating Scale**	115	8.0 (0.0–37.0)	12.0 (0.0–37.0)	8.0 (0.0–30.0)	**0.002**	**0.024**	0.193
**Personal and Social Performance Scale**	115	75.0 (20.0–100.0)	70.0 (20.0–95.0)	77.0 (22.0–100.0)	**0.040**	0.180	0.778
**Beck Cognitive Insight Scale**	87	16.0 (1.0–18.0)	14.0 (1.0–18.0)	18.0 (9.0–18.0)	**<0.001**	**<0.001**	1.387
**Family history of psychiatric diagnosis**	115	52 (45.2%)	16 (40.0%)	36 (48.0%)	0.532	0.799	0.077
**Family history of severe mental illness**	115	17 (14.8%)	3 (7.5%)	14 (18.7%)	0.183	0.412	0.150
**Worked while raising child**	115	31 (27.0%)	8 (20.0%)	23 (30.7%)	0.314	0.605	0.114
**Planned pregnancy**	115	81 (70.4%)	29 (72.5%)	52 (69.3%)	0.889	>0.999	0.033
**Feeling ready for motherhood**	115	80 (69.6%)	32 (80.0%)	48 (64.0%)	0.118	0.321	0.166
**History of custody case**	106	16 (15.1%)	5 (16.1%)	11 (14.7%)	>0.999	>0.999	0.019
**Motherhood effective in treatment adherence**	115	81 (70.4%)	30 (75.0%)	51 (68.0%)	0.569	0.809	0.073
**Education level**	115				0.626	0.835	0.090
*Literate only*		13 (11.3%)	6 (15.0%)	7 (9.3%)			
*Primary school*		76 (66.1%)	26 (65.0%)	50 (66.7%)			
*High school or above*		26 (22.6%)	8 (20.0%)	18 (24.0%)			
**Marital status**	115				0.071	0.275	0.214
*Married*		90 (78.3%)	27 (67.5%)	63 (84.0%)			
*Widowed*		4 (3.5%)	3 (7.5%)	1 (1.3%)			
*Divorced*		21 (18.3%)	10 (25.0%)	11 (14.7%)			
**Employment status**	115				0.122	0.321	0.175
*Unemployed*		104 (90.4%)	39 (97.5%)	65 (86.7%)			
*Employed*		11 (9.6%)	1 (2.5%)	10 (13.3%)			
**Economic status**	115				0.131	0.321	0.188
*Low*		32 (27.8%)	15 (37.5%)	17 (22.7%)			
*Middle*		75 (65.2%)	24 (60.0%)	51 (68.0%)			
*Good*		8 (7.0%)	1 (2.5%)	7 (9.3%)			
**Living arrangement**	115				**0.013**	0.089	0.306
*Nuclear family (spouse and children)*		80 (69.6%)	21 (52.5%)	59 (78.7%)			
*Extended family (spouse, children + others)*		13 (11.3%)	7 (17.5%)	6 (8.0%)			
*Child and mother’s relatives*		14 (12.2%)	6 (15.0%)	8 (10.7%)			
*Only child and mother*		8 (7.0%)	6 (15.0%)	2 (2.7%)			
**Effect of motherhood on coping with illness**	115				0.094	0.318	0.203
*Negative*		27 (23.5%)	11 (27.5%)	16 (21.3%)			
*Neutral*		17 (14.8%)	2 (5.0%)	15 (20.0%)			
*Positive*		71 (61.7%)	27 (67.5%)	44 (58.7%)			

^1^ Continuous variables are presented as median (minimum–maximum); categorical variables as n (%).

^2^ Group comparisons were performed using Wilcoxon rank-sum test for continuous variables and Pearson’s chi-square or Fisher’s exact test for categorical variables, as appropriate.

^3^ Benjamini–Hochberg false discovery rate correction was applied across baseline comparisons.

^4^ Effect size was reported as rank-biserial correlation for continuous comparisons and Cramer’s V for categorical comparisons.

**Abbreviations:** BPRS: Brief Psychiatric Rating Scale; CGI: Clinical Global Impression; FDR: False Discovery Rate; PSP: Personal and Social Performance Scale; SPPR: Self-Perception of Parental Role; SubRAS: Subjective Recovery Assessment Scale.SubRAS Total result was made bold within the text. *p* < 0.05 are in bold.

In the baseline comparisons, SubRAS total scores differed between diagnostic groups in the raw analyses (median 62.0 vs 54.0; raw *p* = 0.013), but this difference did not remain statistically significant after FDR adjustment (FDR-adjusted *p* = 0.089). In contrast, BPRS scores remained significantly higher in the schizophrenia group after correction for multiple comparisons (raw *p* = 0.002; FDR-adjusted *p* = 0.024). BCIS scores were also higher in the BD group (*p* < 0.001). No significant differences were observed in other demographic and motherhood-related variables between the diagnostic groups following adjustment for multiple comparisons.

Correlational analyses indicated a positive association between subjective recovery, as quantified by SubRAS, and several dimensions of parental role perception (Table 2). In the overall sample, SubRAS correlated significantly with SPPR Role Satisfaction (*r* = 0.296), Parental Efficacy (*r* = 0.276), Parental Investment (*r* = 0.331), Role Balance (*r* = 0.240), and SPPR Total Score (*r* = 0.413), all of which remained significant after FDR correction. A positive association was observed between recovery scores and PSP scores (*r* = 0.463) and BCIS scores (*r* = 0.320). In contrast, a negative relationship was found with BPRS scores (*r*=-0.394) and CGI scores (*r*=-0.449). The SCH subgroup demonstrated that SubRAS was significantly positively correlated with parental efficacy, parental investment, total SPPR, personal and social functioning (all *p* < 0.050), and PANSS total and subscale scores, as expected in the inverse direction. Within the BD subgroup, significant positive associations were identified with role satisfaction, role balance, parental investment, total SPPR, and functioning. In contrast, HDRS scores were negatively correlated with SubRAS (*r*=-0.382), YMRS scores were not significantly associated with recovery.

**Table 2.** Correlations of SubRAS with parental role, symptom severity, functioning, and insight variables.

Variable	Overall			Schizophrenia			Bipolar disorder		
	*N*	Spearman’s rho	FDR-adjusted *p*	*N*	Spearman’s rho	FDR-adjusted *p*	*N*	Spearman’s rho	FDR-adjusted *p*
**SPPR**									
*SPPR Role Satisfaction*	115	0.296	**0.002**	40	0.352	**0.031**	75	0.312	**0.009**
*SPPR Parental Efficacy*	115	0.276	**0.004**	40	0.475	**0.008**	75	0.144	0.248
*SPPR Parental Investment*	115	0.331	**<0.001**	40	0.398	**0.016**	75	0.342	**0.004**
*SPPR Role Balance*	115	0.240	**0.010**	40	0.022	0.893	75	0.395	**0.001**
*SPPR Total Score*	115	0.413	**<0.001**	40	0.519	**0.007**	75	0.395	**0.001**
**Clinical Global Impression**	39	−0.449	**0.005**	38	−0.457	**0.012**	–	–	**-**
**Brief Psychiatric Rating Scale**	115	−0.394	**<0.001**	–	–	**-**	–	–	**-**
**Personal and Social Performance Scale**	115	0.463	**<0.001**	40	0.404	**0.016**	75	0.480	**<0.001**
**Beck Cognitive Insight Scale**	87	0.320	**0.004**	40	0.203	0.228	–	–	–
**PANSS**									
*Total*	–	–	–	39	−0.490	**0.008**	–	–	–
*Positive*	–	–	–	40	−0.413	**0.016**	–	–	–
*Negative*	–	–	–	40	−0.418	**0.016**	–	–	–
*General*	–	–	–	40	−0.383	**0.020**	–	–	–
**Young Mania Rating Scale**	–	–	–	–	–	–	74	**0.008**	0.944
**Hamilton Depression Rating Scale**	–	–	–	–	–	–	74	−0.382	**0.002**

Dashes indicate variables not assessed in that diagnostic group. Spearman’s rank correlation coefficients are shown. Benjamini–Hochberg False discovery rate (FDR correction was applied separately within the overall, schizophrenia, and bipolar correlation sets.

**Abbreviations:** BPRS: Brief Psychiatric Rating Scale; CGI: Clinical Global Impression; FDR: False Discovery Rate; HDRS: Hamilton Depression Rating Scale; PANSS: Positive and Negative Syndrome Scale; PSP: Personal and Social Performance Scale; SPPR: Self-Perception of Parental Role; YMRS: Young Mania Rating Scale.*p* < 0.05 are in bold.

Table 3 indicates that experiences concerning motherhood were differentially associated with parental role perception and recovery. The history of custody case was not associated with significant differences in SPPR or SubRAS scores after FDR correction. Perceiving motherhood as beneficial for treatment adherence did not correlate with higher SubRAS scores; however, women endorsing this item had higher SPPR Role Balance scores before correction. Conversely, the perceived effect of motherhood on coping with illness demonstrated a significant relationship with both perceived parental roles and subjective recovery. Significantly higher SPPR Role Satisfaction, Role Balance, SPPR Total, and SubRAS Total scores were reported by women who perceived a positive effect of motherhood on coping, relative to those reporting neutral or negative effects. These associations remained statistically significant following FDR adjustment.

**Table 3.** SubRAS and SPPR scores according to motherhood-related experiences.

History of custody case						
	N	No	Yes	Raw p	FDR-adjusted *p*	Effect size
SPPR Role Satisfaction	106	20.5 (7.0–25.0)	17.0 (9.0–25.0)	0.094	0.283	0.262
SPPR Parental Efficacy	106	21.0 (8.0–30.0)	16.5 (7.0–27.0)	**0.021**	0.127	0.363
SPPR Parental Investment	106	14.0 (5.0–26.0)	14.0 (10.0–21.0)	0.986	0.986	0.003
SPPR Role Balance	106	21.0 (8.0–30.0)	21.0 (8.0–30.0)	0.482	0.578	0.111
SPPR Total	106	77.0 (41.0–106.0)	74.0 (47.0–97.0)	0.220	0.439	0.194
SubRAS Total	106	60.0 (20.0–87.0)	50.0 (30.0–84.0)	0.345	0.517	0.149

*p* < 0.05 are in bold

**Table:** 

Motherhood effective in treatment adherence						
	N	No	Yes	Raw p	FDR-adjusted *p*	Effect size
SPPR Role Satisfaction	115	19.5 (9.0–25.0)	21.0 (7.0–25.0)	0.235	0.340	0.572
SPPR Parental Efficacy	115	19.5 (7.0–30.0)	21.0 (7.0–29.0)	0.849	0.849	0.455
SPPR Parental Investment	115	14.0 (9.0–25.0)	15.0 (5.0–26.0)	0.259	0.340	0.566
SPPR Role Balance	115	19.0 (8.0–30.0)	22.0 (8.0–30.0)	**0.019**	0.112	0.711
SPPR Total	115	73.0 (41.0–101.0)	78.0 (47.0–106.0)	0.094	0.282	0.631
SubRAS Total	115	55.5 (28.0–85.0)	62.0 (20.0–87.0)	0.283	0.340	0.560

*p* < 0.05 are in bold

**Table:** 

Effect of motherhood on coping with illness							
	N	Negative	Neutral	Positive	Raw p	FDR-adjusted *p*	Effect size
SPPR Role Satisfaction	115	19.0 (9.0–25.0)	18.0 (11.0–25.0)	21.0 (7.0–25.0)	**0.031**	**0.047**	0.044
SPPR Parental Efficacy	115	19.0 (7.0–28.0)	21.0 (13.0–30.0)	21.0 (9.0–30.0)	0.538	0.538	0.000
SPPR Parental Investment	115	14.0 (9.0–25.0)	14.0 (10.0–19.0)	16.0 (5.0–26.0)	0.077	0.093	0.028
SPPR Role Balance	115	20.0 (9.0–28.0)	18.0 (8.0–28.0)	23.0 (8.0–30.0)	**<0.001**	**0.005**	0.110
SPPR Total	115	75.0 (41.0–97.0)	73.0 (52.0–95.0)	81.0 (48.0–106.0)	**0.007**	**0.014**	0.071
SubRAS Total	115	50.0 (20.0–80.0)	55.0 (28.0–87.0)	63.0 (23.0–85.0)	**0.002**	**0.006**	0.092

Values are presented as median (minimum–maximum). Wilcoxon rank-sum test was used for two-group comparisons; Kruskal–Wallis test was used for three-group comparisons. Effect size: rank-biserial correlation for two-group comparisons; eta-squared (H) for three-group comparisons. False discovery rate (FDR) adjustment was applied within each analysis panel.

**Abbreviations:** FDR: False Discovery Rate; SPPR: Self-Perception of Parental Role; SubRAS: Subjective Recovery Assessment Scale.SubRAS Total result was made bold within the text. *p* < 0.05 are in bold

As shown in Table 4, SubRAS scores differed significantly based on diagnosis, economic status, education level, and perceived effect of motherhood on coping with illness after FDR correction. Recovery scores were higher for women diagnosed with BD, individuals with higher education, and those reporting a positive effect of motherhood on coping. No significant differences were found for marital status, employment, family psychiatric history, custody case history, number of children, or treatment adherence item after correction. FDR adjustments were applied separately within different families of statistical comparisons. Baseline comparisons presented in Table 1 and SubRAS-related inferential analyses presented in Table 4 were corrected independently. Therefore, FDR-adjusted p-values are not directly comparable across these tables.

**Table 4.** Differences in SubRAS scores across demographic, socioeconomic, and motherhood-related groups.

Variable	*N*	Value	Raw *p*	FDR-adjusted *p*	Effect size
**Demographic and socioeconomic characteristics**					
**Diagnosis**	115		**0.013**	**0.046**	0.828
*Schizophrenia*		54.0 (23.0–84.0)			
*Bipolar disorder*		62.0 (20.0–87.0)			
**Education level**	115		**0.020**	**0.047**	0.052
*Literate only*		38.0 (28.0–67.0)			
*Primary school*		59.0 (20.0–87.0)			
*High school or above*		62.0 (25.0–85.0)			
**Marital status**	115		0.096	0.153	0.024
*Married*		60.0 (20.0–87.0)			
*Widowed*		35.0 (23.0–70.0)			
*Divorced*		50.0 (30.0–84.0)			
**Employment status**	115		0.746	0.746	9.606
*Unemployed*		59.0 (20.0–87.0)			
*Employed*		63.0 (48.0–71.0)			
**Economic status**	115		**0.010**	**0.046**	0.065
*Low*		52.5 (23.0–85.0)			
*Middle*		63.0 (20.0–87.0)			
*Good*		40.5 (26.0–67.0)			
**Family history of psychiatric diagnosis**	115		0.109	0.153	1.056
*No*		60.0 (28.0–87.0)			
*Yes*		58.0 (20.0–84.0)			
**Family history of severe mental illness**	115		0.699	0.746	5.764
*No*		59.0 (23.0–87.0)			
*Yes*		54.0 (20.0–80.0)			
**Motherhood-related characteristics**					
**Worked while raising child**	115		0.215	0.431	2.893
*No*		59.0 (20.0–87.0)			
*Yes*		63.0 (39.0–77.0)			
**Planned pregnancy**	115		0.074	0.297	0.644
*No*		52.5 (29.0–87.0)			
*Yes*		63.0 (20.0–85.0)			
**Feeling ready for motherhood**	115		0.740	0.740	0.489
*No*		58.0 (20.0–85.0)			
*Yes*		60.0 (23.0–87.0)			
**Living arrangement**	115		0.211	0.431	0.014
*Nuclear family (spouse and children)*		60.0 (20.0–87.0)			
*Extended family (spouse, children + others)*		55.0 (32.0–84.0)			
*Child and mother’s relatives*		50.0 (30.0–79.0)			
*Only child and mother*		44.5 (23.0–68.0)			
**Number of children**	115		0.575	0.657	0.000
*1 child*		60.5 (32.0–85.0)			
*2 children*		59.0 (25.0–82.0)			
*3+ children*		60.0 (20.0–87.0)			
**History of custody case**	106		0.345	0.460	5.538
*No*		60.0 (20.0–87.0)			
*Yes*		50.0 (30.0–84.0)			
**Motherhood effective in treatment adherence**	115		0.283	0.453	0.560
*No*		55.5 (28.0–85.0)			
*Yes*		62.0 (20.0–87.0)			
**Effect of motherhood on coping with illness**	115		**0.002**	**0.017**	0.092
*Negative*		50.0 (20.0–80.0)			
*Neutral*		55.0 (28.0–87.0)			
*Positive*		63.0 (23.0–85.0)			

Values are presented as median (minimum–maximum). Wilcoxon rank-sum test was used for two-group comparisons; Kruskal–Wallis test was used for comparisons across three or more groups. Effect size: rank-biserial correlation for two-group comparisons; eta-squared (H) for comparisons across three or more groups. Benjamini–Hochberg False discovery rate (FDR) correction was applied across all comparisons. FDR correction was applied within the set of SubRAS-related inferential comparisons presented in this table. This correction family differs from the baseline comparisons reported in Table 1; therefore, FDR-adjusted p-values are not directly comparable across tables.

**Abbreviations:** FDR: False Discovery Rate.*p* < 0.05 are in bold.

The multivariable linear regression analyses are detailed in Table 5. In the overall sample, greater SPPR parental investment (*B* = 0.823, *p* = 0.049) and lower BPRS scores (B = −0.398, *p* = 0.028) were independently associated with higher SubRAS scores. BCIS scores showed a positive association with recovery scores (*B* = 0.786, *p* = 0.050). Parental efficacy, functioning, and education level were retained in the final model but did not reach statistical significance. The model accounted for 33.0% of the variability in SubRAS scores (adjusted R^2^=0.330, model *p* < 0.001). In the SCH subgroup, parental efficacy (*B* = 1.558, *p* < 0.001), parental investment (*B* = 1.410, *p* = 0.005), and cognitive insight (*B* = 0.994, *p* = 0.046) were independent positive predictors of recovery, explaining 40.3% of the variance. In the BD subgroup, parental investment (*B* = 1.071, *p* = 0.042) and personal and social functioning (*B* = 0.242, *p* = 0.034) were positive predictors, whereas depressive symptom severity was a negative predictor (B=-0.846, *p* = 0.022), with the model explaining 22.9% of the variance. The multivariable regression models were considered the main inferential analyses of the study, whereas subgroup comparisons and motherhood-related group analyses were interpreted as exploratory and hypothesis-generating. Sensitivity analyses revealed consistent patterns regardless of model variations, and low VIF values indicated strong robustness and stability in the regression models (Supplementary Tables S2 and S3). Univariable associations are additionally presented in Supplementary Table S4.

**Table 5.** Multivariable linear regression models for SubRAS scores in the overall sample and diagnostic subgroups.

Predictor	B	SE	Standardized Beta	95% CI	*p*-value	VIF
**Model 1. Overall sample** *(N = 87; Adjusted R²=0.330; F = 8.07; Model p= <0.001)*						
SPPR Parental Efficacy	0.419	0.292	0.143	−0.162 to 1.000	0.155	1.270
SPPR Parental Investment	0.823	0.411	0.192	0.005 to 1.642	**0.049**	1.190
Brief Psychiatric Rating Scale	−0.398	0.178	−0.233	−0.752 to −0.045	**0.028**	1.380
Personal and Social Performance Scale	0.165	0.101	0.157	−0.037 to 0.367	0.107	1.200
Beck Cognitive Insight Scale	0.786	0.394	0.187	0.002 to 1.570	0.050	1.130
Education level	5.324	2.851	0.186	−0.350 to 10.997	0.066	1.280
**Model 2. Schizophrenia subgroup** *(N = 39; Adjusted R²=0.403; F = 9.55; Model p= <0.001)*						
SPPR Parental Efficacy	1.558	0.386	0.510	0.774 to 2.342	**<0.001**	1.020
SPPR Parental Investment	1.410	0.465	0.381	0.467 to 2.353	**0.005**	1.000
Beck Cognitive Insight Scale	0.994	0.480	0.262	0.019 to 1.969	**0.046**	1.020
**Model 3. Bipolar disorder subgroup** *(N = 74; Adjusted R²=0.229; F = 8.23; Model p= <0.001)*						
SPPR Parental Investment	1.071	0.516	0.220	0.042 to 2.100	**0.042**	1.060
Hamilton Depression Rating Scale	−0.846	0.360	−0.261	−1.564 to −0.128	**0.022**	1.170
Personal and Social Performance Scale	0.242	0.112	0.238	0.018 to 0.465	**0.034**	1.150

*Dependent variable: SubRAS Total score. Backward elimination based on Akaike information criterion (AIC) was used to derive the final models. Standardized beta coefficients are reported alongside unstandardized coefficients. Variance inflation factor (VIF) values are shown for predictors retained in the final models*.

**Abbreviations:** AIC: Akaike Information Criterion; BPRS: Brief Psychiatric Rating Scale; CI: Confidence Interval; HDRS: Hamilton Depression Rating Scale; SE: Standard Error; SPPR: Self-Perception of Parental Role; SubRAS: Subjective Recovery Assessment Scale; VIF: Variance Inflation Factor.*p* < 0.05 are in bold

## Discussion

The present study investigated the relationship between perceptions of the motherhood role and subjective motivation for recovery in women diagnosed with severe mental illnesses, specifically SCH and BD. Overall, the findings of the study highlighted that more positive perceptions of the parental role were associated with higher levels of subjective recovery motivation. Across all participants, parental investment emerged as an independent factor associated with recovery motivation, but the different dimensions of the maternal role played a more significant part within different diagnostic groups. In women with SCH, parental competence and investment showed a particular relationship with recovery motivation, while parental role satisfaction was more strongly associated to recovery motivation among women with BD. These findings suggest that motherhood-related identity and role perceptions may be associated with subjective recovery motivation among women with severe mental illness.

Motherhood is frequently a central component of identity for many women and may function as a meaningful life role even in the context of psychiatric illness [18,19]. Previous studies have revealed that parenting responsibilities could contribute a sense of purpose, social connection, and emotional meaning, which are fundamental to personal recovery [5,6]. The CHIME framework describes recovery as a multidimensional process including connectedness, identity, meaning, hope, and empowerment [5]. Meaningful social roles, such as those associated with parenting, may contribute to several of these domains [5]. In line with this framework, our research indicates that maternal role perceptions, particularly parental investment, were associated with a stronger sense of purpose and higher subjective recovery motivation.

Parental investment emerged as the most significant predictor of recovery motivation across the entire sample when considering multivariable analyses. The commitment and emotional involvement in parenting, as well as the significance of caregiving, are encapsulated in parental investment [10]. Prior studies have suggested that taking on meaningful social roles may strengthen resilience and promote recovery-focused behaviors for individuals experiencing severe mental illness [20]. Our findings are consistent with the view that greater psychological engagement in parenting is associated with higher recovery motivation.

Interestingly, the associations between maternal role perceptions and recovery motivation were not consistent among the different diagnostic groups. For women with SCH, recovery motivation was significantly related to parental competence and parental investment. These findings are consistent with previous studies suggesting that parenting responsibilities can foster a sense of competence, responsibility, and social value in individuals with serious mental illness [1,2]. Perceived parental competence may be related to greater engagement in treatment and rehabilitation activities [2,8]. Within this framework, motherhood presents a possibility for exhibiting proficiency and assuming a socially valuable position, which could positively impact recovery.

Conversely, for women diagnosed with BD, the degree of parental role satisfaction demonstrated a stronger correlation with subjective recovery motivation. Parental satisfaction reflects emotional gratification and positive effects derived from interactions with one’s child. Given that BD is often characterized by fluctuations in mood and emotional experience, positive interpersonal interactions within family relationships may be associated with higher perceived recovery [21,22]. Studies on recovery in BD have previously underscored that the process is significantly shaped by the meaning-making of individual, identity reconstruction, and valued life roles in shaping recovery experiences [6,23]. This perspective is supported by the results of the current study, which suggest that women with BD might find emotional satisfaction within the parenting role particularly beneficial for recovery processes.

Clinical and functional determinants, beyond parenting perceptions, also correlated with recovery motivation. A positive association was observed between higher subjective recovery scores and improved functioning and lower psychiatric symptom severity in all participants. Consistent with previous studies, these results indicate that symptom burden and functional impairment continue to be pivotal factors influencing recovery outcomes in severe mental illness [24,25]. Despite personal recovery encompassing more than symptom remission, clinical stability and social functioning remain significant factors in influencing individuals’ recovery experiences [23].

The present findings also highlight the potential role of cognitive insight in recovery processes. Cognitive insight demonstrated a positive association with recovery motivation among individuals in the SCH subgroup. Previously, enhanced understanding of illness has been associated with better treatment adherence, improved psychosocial functioning, and more favorable long-term outcomes in severe mental illness [25–27]. Greater cognitive insight has been associated with better understanding of illness and more active engagement in recovery-oriented behaviors [28].

Furthermore, this investigation revealed that a significant number of individuals believed that becoming a mother enhanced their capacity to manage mental health challenges. Women who reported that motherhood positively influenced coping with illness had significantly higher parental role satisfaction and overall recovery scores. Similarly, previous studies highlighted that the responsibilities of parenting may be a significant motivator for individuals with severe mental illness to pursue treatment and maintain stability in order to care for their children [1,2]. These findings suggest that motherhood may represent an important role associated with greater recovery motivation.

From a clinical perspective, these findings may also have implications for recovery-oriented mental health services [2,8,20]. Community-based care may benefit from prevention-oriented approaches that identify parenting-related challenges early and provide coordinated psychosocial and social support. When adequate support systems are available, motherhood may strengthen meaning, responsibility, and motivation for recovery. However, when caregiving demands exceed available resources, the same role may increase stress and undermine parental self-efficacy. Integrating family-oriented and community-supported interventions into recovery-oriented services may therefore help support both parenting roles and recovery processes.

Nevertheless, the connection between motherhood and mental illness is complicated and should not be viewed exclusively in positive terms. Parenting responsibilities may impose substantial caregiving stress, emotional distress, and role strain, particularly in the presence of ongoing symptoms, financial difficulties, or insufficient social networks [7,29]. Prior research has indicated difficulties including custody concerns, stigma, apprehension regarding child custody, and parenting-related stress among women with severe mental illness [3]. Therefore, motherhood may function both as a source of meaning and as a potential stressor, and recovery-oriented services should consider both dimensions when supporting mothers with psychiatric disorders [2,30].

This study has several limitations that should be considered when interpreting the findings. The cross-sectional nature of the study precludes causal conclusions about the link between maternal role perceptions and motivation for recovery. It is therefore unclear whether positive parenting perceptions contribute to higher recovery motivation or whether individuals with greater recovery motivation perceive their parenting roles more positively. Second, disparities in the sizes of the diagnostic groups, characterized by a lesser number of individuals in the SCH subgroup, could have reduced statistical power for subgroup analyses and increased the possibility of type II errors. The exclusion of a comparable group of non-maternal women with severe mental illness restricted the ability to isolate the specific contribution of motherhood to recovery processes. Additionally, the SubRAS has been validated in individuals with SCH [11]; however, its validation in populations with BD has not been thoroughly conducted. Finally, several potentially relevant contextual factors—such as partner support, caregiving involvement, and detailed custody arrangements—were not available in the dataset and therefore could not be included in the analysis. By including more extensive family and social context variables in future longitudinal studies, researchers may provide a deeper understanding of the connection between motherhood and recovery in severe mental illness. Therefore, findings from subgroup and motherhood-related categorical analyses should be interpreted as exploratory and hypothesis-generating rather than confirmatory.

Despite these limitations, the study also has several strengths. To the best of our knowledge, this research represents one of a limited number of studies that specifically investigate the connection between the perception of the motherhood role and an individual’s subjective motivation for recovery in women diagnosed with severe mental illness. By incorporating psychosocial role perceptions with clinical and functional evaluations, the current study facilitates a more holistic understanding of recovery processes. In addition, the integration of validated instruments for psychiatric assessment, alongside the incorporation of women engaged in community mental health services, enhance the clinical relevance of the findings. By focusing on motherhood as a meaningful social role, the study contributes to the growing literature emphasizing the importance of identity and life roles within recovery-oriented mental health care.

## Conclusion

In this study, the association between motherhood-related role perceptions and subjective recovery motivation was observed in women with severe mental illness. Parental investment, competence, and satisfaction were positively associated with higher recovery scores. Variations in the patterns of associations across diagnostic groups suggest that motherhood-related role perceptions may be linked to recovery motivation in different ways in schizophrenia and bipolar disorder. These findings emphasize the critical need to acknowledge essential social roles, such as motherhood, within recovery-oriented mental health care. For women with severe mental illness, the parenting role may include both supportive and challenging aspects. Future longitudinal studies are needed to clarify the causal relationships between parenting roles and recovery processes.

## Supplementary Material

supplement.docx

## Data Availability

The data that support the findings of this study are available from the corresponding author upon reasonable request.
