# Supplementary material for: The impact of the motherhood role on recovery motivation in women with severe mental illness
Source: Ann Med. 2026 Aug 18;58(1):2714593. doi: 10.1080/07853890.2026.2714593 (PMC13488503; doi:10.1080/07853890.2026.2714593)
Supplement: Supplementary file 1 [file IANN_A_2714593_SM8749.docx]

# Supplementary Table S1. Description of clinical and psychometric scales used in the study

| **Scale** | **Construct Measured** | **Items** | **Score Range** | **Higher Score Indicates** | **Study Population** |
| --- | --- | --- | --- | --- | --- |
| SPPR | Perceived parental role (satisfaction, competence/efficacy, investment, role balance) | 22 | 1–5 per item | More positive parental role perception | All participants |
| SubRAS | Subjective recovery motivation | 17 | 17–85 | Better subjective recovery | All participants |
| BPRS | General psychiatric symptom severity | 18 | 1–7 per item | More severe psychiatric symptoms | All participants |
| PANSS | Positive, negative, and general psychopathology symptoms | 30 | 30–210 | More severe schizophrenia symptoms | Schizophrenia subgroup |
| YMRS | Mania severity | 11 | 0–60 | More severe manic symptoms | Bipolar disorder subgroup |
| HDRS-17 | Depression severity | 17 | 0–52 | More severe depressive symptoms | Bipolar disorder subgroup |
| PSP | Social and personal functioning | 4 domains | 1–100 | Better functioning | All participants |
| BCIS | Cognitive insight | 15 | 0–45 | Greater cognitive insight | All participants |
| CGI | Overall clinical severity | 1 | 1–7 | Greater clinical severity | All participants |

**Abbreviations:** BCIS, Beck Cognitive Insight Scale; BPRS, Brief Psychiatric Rating Scale; CGI, Clinical Global Impression; HDRS, Hamilton Depression Rating Scale; PANSS, Positive and Negative Syndrome Scale; PSP, Personal and Social Performance Scale; SPPR, Self-Perception of Parental Role Scale; SubRAS, Subjective Recovery Assessment Scale; YMRS, Young Mania Rating Scale.

# Supplementary Table S2. Sensitivity analyses for multivariable linear regression models predicting SubRAS scores

| **Specification** | **Predictor** | **B** | **Std Beta** | **95% CI** | **p-value** | **N** | **Adj R²** | **AIC** |
| --- | --- | --- | --- | --- | --- | --- | --- | --- |
| **Model 1. Overall sample** | | | | | | | | |
| Primary model | SPPR Parental Investment | 1.071 | 0.220 | 0.042 to 2.100 | **0.042** | 74 | 0.229 | 598.000 |
| Primary model | Hamilton Depression Rating Scale | -0.846 | -0.261 | -1.564 to -0.128 | **0.022** | 74 | 0.229 | 598.000 |
| Primary model | Personal and Social Performance Scale | 0.242 | 0.238 | 0.018 to 0.465 | **0.034** | 74 | 0.229 | 598.000 |
| *Primary model* | ***Retained predictors:*** *SPPR Parental Investment, Hamilton Depression Rating Scale, Personal and Social Performance Scale* |  |  |  |  | *74* | *0.229* | *598.000* |
| Sensitivity model 1: without PSP | SPPR Parental Investment | 1.199 | 0.246 | 0.151 to 2.247 | **0.026** | 74 | 0.189 | 600.770 |
| Sensitivity model 1: without PSP | Hamilton Depression Rating Scale | -1.097 | -0.339 | -1.794 to -0.401 | **0.002** | 74 | 0.189 | 600.770 |
| *Sensitivity model 1: without PSP* | ***Retained predictors:*** *SPPR Parental Investment, Hamilton Depression Rating Scale* |  |  |  |  | *74* | *0.189* | *600.770* |
| Sensitivity model 2: without mood symptom scores | SPPR Parental Investment | 1.181 | 0.262 | 0.221 to 2.141 | **0.017** | 75 | 0.182 | 608.850 |
| Sensitivity model 2: without mood symptom scores | Personal and Social Performance Scale | 0.328 | 0.323 | 0.112 to 0.545 | **0.003** | 75 | 0.182 | 608.850 |
| *Sensitivity model 2: without mood symptom scores* | ***Retained predictors:*** *SPPR Parental Investment, Personal and Social Performance Scale* |  |  |  |  | *75* | *0.182* | *608.850* |
| **Model 2. Schizophrenia subgroup** | | | | | | | | |
| Primary model | SPPR Parental Efficacy | 0.419 | 0.143 | -0.162 to 1.000 | 0.155 | 87 | 0.330 | 705.120 |
| Primary model | SPPR Parental Investment | 0.823 | 0.192 | 0.005 to 1.642 | **0.049** | 87 | 0.330 | 705.120 |
| Primary model | Brief Psychiatric Rating Scale | -0.398 | -0.233 | -0.752 to -0.045 | **0.028** | 87 | 0.330 | 705.120 |
| Primary model | Personal and Social Performance Scale | 0.165 | 0.157 | -0.037 to 0.367 | 0.107 | 87 | 0.330 | 705.120 |
| Primary model | Beck Cognitive Insight Scale | 0.786 | 0.187 | 0.002 to 1.570 | 0.050 | 87 | 0.330 | 705.120 |
| Primary model | Education level | 5.324 | 0.186 | -0.350 to 10.997 | 0.066 | 87 | 0.330 | 705.120 |
| *Primary model* | ***Retained predictors:*** *SPPR Parental Efficacy, SPPR Parental Investment, Brief Psychiatric Rating Scale, Personal and Social Performance Scale, Beck Cognitive Insight Scale, Education level* |  |  |  |  | *87* | *0.330* | *705.120* |
| Sensitivity model 1: without PSP | SPPR Parental Efficacy | 0.455 | 0.155 | -0.130 to 1.040 | 0.126 | 87 | 0.316 | 705.960 |
| Sensitivity model 1: without PSP | SPPR Parental Investment | 0.895 | 0.209 | 0.072 to 1.717 | **0.033** | 87 | 0.316 | 705.960 |
| Sensitivity model 1: without PSP | Brief Psychiatric Rating Scale | -0.472 | -0.276 | -0.817 to -0.126 | **0.008** | 87 | 0.316 | 705.960 |
| Sensitivity model 1: without PSP | Beck Cognitive Insight Scale | 0.900 | 0.214 | 0.121 to 1.680 | **0.024** | 87 | 0.316 | 705.960 |
| Sensitivity model 1: without PSP | Education level | 5.197 | 0.182 | -0.531 to 10.924 | 0.075 | 87 | 0.316 | 705.960 |
| *Sensitivity model 1: without PSP* | ***Retained predictors:*** *SPPR Parental Efficacy, SPPR Parental Investment, Brief Psychiatric Rating Scale, Beck Beck Cognitive Insight Scale, Education level* |  |  |  |  | *87* | *0.316* | *705.960* |
| Sensitivity model 2: without BPRS | SPPR Parental Efficacy | 0.655 | 0.223 | 0.101 to 1.210 | **0.021** | 87 | 0.297 | 708.420 |
| Sensitivity model 2: without BPRS | SPPR Parental Investment | 0.902 | 0.211 | 0.067 to 1.738 | **0.035** | 87 | 0.297 | 708.420 |
| Sensitivity model 2: without BPRS | Personal and Social Performance Scale | 0.223 | 0.212 | 0.023 to 0.423 | **0.029** | 87 | 0.297 | 708.420 |
| Sensitivity model 2: without BPRS | Beck Cognitive Insight Scale | 0.912 | 0.217 | 0.117 to 1.707 | **0.025** | 87 | 0.297 | 708.420 |
| Sensitivity model 2: without BPRS | Education level | 6.469 | 0.226 | 0.752 to 12.186 | **0.027** | 87 | 0.297 | 708.420 |
| *Sensitivity model 2: without BPRS* | ***Retained predictors:*** *SPPR Parental Efficacy, SPPR Parental Investment, Personal and Social Performance Scale, Beck Cognitive Insight Scale, and Education level* |  |  |  |  | *87* | *0.297* | *708.420* |
| **Model 3. Bipolar disorder subgroup** | | | | | | | | |
| Primary model | SPPR Parental Efficacy | 1.558 | 0.510 | 0.774 to 2.342 | **<0.001** | 39 | 0.403 | 311.870 |
| Primary model | SPPR Parental Investment | 1.410 | 0.381 | 0.467 to 2.353 | **0.005** | 39 | 0.403 | 311.870 |
| Primary model | Beck Cognitive Insight Scale | 0.994 | 0.262 | 0.019 to 1.969 | **0.046** | 39 | 0.403 | 311.870 |
| *Primary model* | ***Retained predictors:*** *SPPR Parental Efficacy, SPPR Parental Investment, and Beck Cognitive Insight Scale* |  |  |  |  | *39* | *0.403* | *311.870* |
| Sensitivity model 1: without PSP | SPPR Parental Efficacy | 1.558 | 0.510 | 0.774 to 2.342 | **<0.001** | 39 | 0.403 | 311.870 |
| Sensitivity model 1: without PSP | SPPR Parental Investment | 1.410 | 0.381 | 0.467 to 2.353 | **0.005** | 39 | 0.403 | 311.870 |
| Sensitivity model 1: without PSP | Beck Cognitive Insight Scale | 0.994 | 0.262 | 0.019 to 1.969 | **0.046** | 39 | 0.403 | 311.870 |
| *Sensitivity model 1: without PSP* | ***Retained predictors:*** *SPPR Parental Efficacy, SPPR Parental Investment, and Beck Cognitive Insight Scale* |  |  |  |  | *39* | *0.403* | *311.870* |
| Sensitivity model 2: without PANSS Total | SPPR Parental Efficacy | 1.484 | 0.511 | 0.751 to 2.217 | **<0.001** | 40 | 0.401 | 318.980 |
| Sensitivity model 2: without PANSS Total | SPPR Parental Investment | 1.460 | 0.401 | 0.543 to 2.378 | **0.003** | 40 | 0.401 | 318.980 |
| Sensitivity model 2: without PANSS Total | Beck Cognitive Insight Scale | 0.929 | 0.249 | -0.009 to 1.868 | 0.052 | 40 | 0.401 | 318.980 |
| *Sensitivity model 2: without PANSS Total* | ***Retained predictors:*** *SPPR Parental Efficacy, SPPR Parental Investment, and Beck Cognitive Insight Scale* |  |  |  |  | *40* | *0.401* | *318.980* |

*Sensitivity analyses were conducted using alternative predictor specifications while maintaining the same backward elimination strategy. Dependent variable: SubRAS Total score. Adjusted R² and AIC are reported to facilitate comparison of model stability across specifications.*

**Abbreviations:** BPRS, Brief Psychiatric Rating Scale; CI, Confidence Interval; HDRS, Hamilton Depression Rating Scale; PSP, Personal and Social Performance Scale; SE, Standard Error; SPPR, Self-Perception of Parental Role Scale; SubRAS, Subjective Recovery Assessment Scale.

# Supplementary Table S3. Multicollinearity diagnostics for final regression models

| **Model** | **Predictor** | **VIF** | **Tolerance** |
| --- | --- | --- | --- |
| **Model 1. Overall sample** | SPPR Parental Efficacy | 1.269 | 0.788 |
| **Model 1. Overall sample** | SPPR Parental Investment | 1.186 | 0.843 |
| **Model 1. Overall sample** | Brief Psychiatric Rating Scale | 1.384 | 0.723 |
| **Model 1. Overall sample** | Personal and Social Performance Scale | 1.196 | 0.836 |
| **Model 1. Overall sample** | Beck Cognitive Insight Scale | 1.128 | 0.886 |
| **Model 1. Overall sample** | Education level | 1.278 | 0.782 |
| **Model 2. Schizophrenia subgroup** | SPPR Parental Efficacy | 1.020 | 0.981 |
| **Model 2. Schizophrenia subgroup** | SPPR Parental Investment | 1.003 | 0.997 |
| **Model 2. Schizophrenia subgroup** | Beck Cognitive Insight Scale | 1.023 | 0.978 |
| **Model 3. Bipolar disorder subgroup** | SPPR Parental Investment | 1.063 | 0.941 |
| **Model 3. Bipolar disorder subgroup** | Hamilton Depression Rating Scale | 1.171 | 0.854 |
| **Model 3. Bipolar disorder subgroup** | Personal and Social Performance Scale | 1.154 | 0.866 |

*Tolerance calculated as 1/VIF.*

**Abbreviations:** BPRS, Brief Psychiatric Rating Scale; HDRS, Hamilton Depression Rating Scale; PSP, Personal and Social Performance Scale; SPPR, Self-Perception of Parental Role Scale; VIF, Variance Inflation Factor.

# Supplementary Table S4. Univariable linear associations with SubRAS Total score

| **Predictor** | **Univariable beta** | **95% CI** | **p-value** | **N** |
| --- | --- | --- | --- | --- |
| SPPR Role Satisfaction | 0.979 | 0.339 to 1.618 | **0.003** | 115 |
| SPPR Parental Efficacy | 0.818 | 0.300 to 1.337 | **0.002** | 115 |
| SPPR Parental Investment | 1.409 | 0.664 to 2.154 | **<0.001** | 115 |
| SPPR Role Balance | 0.478 | -0.043 to 0.999 | 0.072 | 115 |
| Brief Psychiatric Rating Scale | -0.736 | -1.018 to -0.453 | **<0.001** | 115 |
| Personal and Social Performance Scale | 0.416 | 0.246 to 0.587 | **<0.001** | 115 |
| Beck Cognitive Insight Scale | 1.301 | 0.439 to 2.163 | **0.003** | 87 |
| Age (years) | -0.184 | -0.474 to 0.106 | 0.211 | 115 |
| Education level | 5.940 | 1.000 to 10.879 | **0.019** | 115 |
| PANSS Total | -0.296 | -0.506 to -0.086 | **0.007** | 39 |
| Young Mania Rating Scale | 0.156 | -0.913 to 1.225 | 0.772 | 74 |
| Hamilton Depression Rating Scale | -1.269 | -1.969 to -0.569 | **<0.001** | 74 |

**Abbreviations:** BPRS, Brief Psychiatric Rating Scale; CI, Confidence Interval; HDRS, Hamilton Depression Rating Scale; PANSS, Positive and Negative Syndrome Scale; PSP, Personal and Social Performance Scale; SPPR, Self-Perception of Parental Role Scale; YMRS, Young Mania Rating Scale.
